# Acute Effects of Static Stretching on Muscle Strength and Power: An Attempt to Clarify Previous Caveats

**DOI:** 10.3389/fphys.2019.01468

**Published:** 2019-11-29

**Authors:** Helmi Chaabene, David G. Behm, Yassine Negra, Urs Granacher

**Affiliations:** ^1^Division of Training and Movement Sciences, Research Focus Cognitive Sciences, University of Potsdam, Potsdam, Germany; ^2^High Institute of Sports and Physical Education, Kef, University of Jendouba, Jendouba, Tunisia; ^3^School of Human Kinetics and Recreation, Memorial University of Newfoundland, St. John’s, NL, Canada; ^4^Research Unit (UR17JS01), Sport Performance, Health and Society, Higher Institute of Sport and Physical Education of Ksar Saîd, University of “La Manouba”, Manouba, Tunisia

**Keywords:** passive stretching, physical fitness, physiology, range of motion, injury

## Abstract

The effects of static stretching (StS) on subsequent strength and power activities has been one of the most debated topics in sport science literature over the past decades. The aim of this review is (1) to summarize previous and current findings on the acute effects of StS on muscle strength and power performances; (2) to update readers’ knowledge related to previous caveats; and (3) to discuss the underlying physiological mechanisms of short-duration StS when performed as single-mode treatment or when integrated into a full warm-up routine. Over the last two decades, StS has been considered harmful to subsequent strength and power performances. Accordingly, it has been recommended not to apply StS before strength- and power-related activities. More recent evidence suggests that when performed as a single-mode treatment or when integrated within a full warm-up routine including aerobic activity, dynamic-stretching, and sport-specific activities, short-duration StS (≤60 s per muscle group) trivially impairs subsequent strength and power activities (∆1–2%). Yet, longer StS durations (>60 s per muscle group) appear to induce substantial and practically relevant declines in strength and power performances (∆4.0–7.5%). Moreover, recent evidence suggests that when included in a full warm-up routine, short-duration StS may even contribute to lower the risk of sustaining musculotendinous injuries especially with high-intensity activities (e.g., sprint running and change of direction speed). It seems that during short-duration StS, neuromuscular activation and musculotendinous stiffness appear not to be affected compared with long-duration StS. Among other factors, this could be due to an elevated muscle temperature induced by a dynamic warm-up program. More specifically, elevated muscle temperature leads to increased muscle fiber conduction-velocity and improved binding of contractile proteins (actin, myosin). Therefore, our previous understanding of harmful StS effects on subsequent strength and power activities has to be updated. In fact, short-duration StS should be included as an important warm-up component before the uptake of recreational sports activities due to its potential positive effect on flexibility and musculotendinous injury prevention. However, in high-performance athletes, short-duration StS has to be applied with caution due to its negligible but still prevalent negative effects on subsequent strength and power performances, which could have an impact on performance during competition.

## Introduction

There is historical tradition saying that stretching has been practiced for thousands of years, mostly by warriors before combat (Behm, 2018). We do not know the preferred stretching technique during the early days; however, today four main stretching techniques (i.e., static, dynamic, ballistic, proprioceptive neuromuscular facilitation) are applied in athletic, fitness, and rehabilitation settings. Specifically, static stretching (StS) involves a controlled continuous movement to the end range-of-motion (ROM) of a single joint or multiple joints by either actively contracting the agonist muscles (i.e., active static) or by using external forces such as gravity, partner, stretching aids (i.e., passive static with stretch bands) (Behm et al., 2016). In the end position, the individual holds the muscle in a lengthening position for a certain time (Behm et al., 2016). Even though StS has widespread usage, it is also the most controversially debated technique with constantly changing views on its positive and negative effects on muscle strength and power.

The general belief that spread from the World Wars until the 1990s is that StS promoted flexibility and improved athletic performance (Behm, 2018). This was mainly substantiated by the thought that greater ROM reduces resistance to movement and improves movement economy (Behm, 2018). However, since the late 1990s up to early 2000s, researchers have started discussing the potential harmful effects of StS on subsequent strength- and power-related activities (Behm et al., 2001; Cornwell et al., 2002; Shrier, 2004a; Wallmann et al., 2005; Young et al., 2006; Cramer et al., 2007; Kay and Blazevich, 2008; McHugh and Nesse, 2008; Behm, 2018; Opplert and Babault, 2018). As a result, it has been widely recommended to avoid performing prolonged StS before strength- and power-related tasks (Magnusson and Renström, 2006; Garber et al., 2011; Simic et al., 2013) and to favor dynamic stretching exercises instead (Behm, 2018). More recently, new evidence on StS challenged the view that it should not be conducted pre-exercise (Behm et al., 2016). In fact, findings from two comprehensive systematic reviews demonstrated that short-duration acute StS (≤60 s) has trivial negative effects on measures of strength and power as opposed to prolonged StS (>60 s) (Kay and Blazevich, 2012; Behm et al., 2016). More recent findings demonstrated that when included in a full warm-up routine, short-duration StS does not impair subsequent strength and power performances (Blazevich et al., 2018; Reid et al., 2018).

The question arises as to the underlying mechanisms responsible for StS-induced impairments in subsequent strength and power activities. Among others, increased compliance of the musculotendinous unit (MTU) that lowers MTU stiffness has been discussed (Avela et al., 1999; Rubini et al., 2007) together with lower motor unit activation (Hough et al., 2009; Trajano et al., 2013). However, the underlying physiological mechanisms following short-duration StS (≤60 s per muscle group) when performed as a single-mode treatment or when integrated into a full warm-up routine have hardly been discussed in the literature (Behm et al., 2016; Lima et al., 2019).

It seems that the contradictory and constantly changing reports with regard to StS research may cause confusion, particularly with coaches and practitioners. It is noteworthy that the acute effects of StS on ROM and physical fitness have recently been discussed (Lima et al., 2019), yet without providing insight into the underlying physiological mechanisms. Therefore, the aims of this review were (1) to provide an overview of previous and current findings on the acute effects of StS on muscle strength and power in physically active individuals and athletes; (2) to update readers’ knowledge related to previous caveats; and (3) to discuss the underlying physiological mechanisms of short-duration StS when performed as a single-mode treatment or when integrated into a full warm-up routine. Information from this review may enable readers to better understand the development of StS research and to refresh their knowledge related to previous controversies. In the end, the authors provide cohort-specific (e.g., high-performance vs. recreational athletes) information based on the current state of the art on the acute effects of StS on muscle strength and power as well as possible avenues for future research.

## Methods

This review included studies that examined the acute effects of StS on subsequent strength and power performances. An electronic literature search was conducted by two authors (HC and YN) in the databases; Medline, ScienceDirect, and Google Scholar. The following key words were used either separately or combined: static stretching, chronic effects, physical performance, strength, power, and injury.

### Inclusion Criteria

Only studies that fulfilled the following inclusion criteria were included in this review: (1) the study addressed a research question related to the acute effects of StS on strength and power performances and (2) included healthy active or competitive individuals (studies conducted with seniors were excluded); (3) the main outcome was a performance or physiological measure; and (4) original or review study written in English and published in a peer-reviewed journal.

### Static Stretching in Disrepute: Acute Effects on Muscle Strength and Power

Pre-exercise StS for the purpose of strength and power performance improvements has widely been criticized (Pope et al., 2000; Shrier, 2004a,b; McHugh and Cosgrave, 2010; Simic et al., 2013). In fact, a large body of scientific evidence has recommended not to use StS immediately before the performance of strength- and power-related activities (Shrier, 2004a; McHugh and Cosgrave, 2010; Simic et al., 2013; Peck et al., 2014). This is based on evidence showing acute stretch-related declines in muscle strength and power. For instance, Fowles et al. (2000) examined the acute effects of StS of the plantar flexors (13 sets of 135 s each with a total of 30 min of time under stretch) on muscle strength (i.e., maximal voluntary contraction) in recreationally active young men aged 22.3 years and women aged 20.3 years. These authors demonstrated significant decreases in maximal isometric voluntary contraction immediately post (∆28%) and at 5 (∆21%), 15 (∆13%), 30 (∆12%), 45 (∆10%), and 60 (∆9%) min after StS. Likewise, Behm and Kibele (2007) studied the effects of four sets of 30 s each with 30 s of rest of different StS intensities (i.e., 50, 75, and 100% of point of discomfort) of the quadriceps, hamstrings, and plantar flexors on jump height performance in physically active male (27.6 years) and female (24 years) university students. The same authors reported significant decrements in jump height (∆3.5%), irrespective of the applied StS intensity. In a systematic literature review, Shrier (2004a) concluded that StS could be harmful to subsequent strength and power actions. In another systematic review, Behm and Chaouachi (2011) suggested that StS has to be implemented with caution if high-speed, power-related activities are required from high-performance athletes. Simic et al. (2013) conducted a meta-analysis including 104 studies that examined the effects of pre-exercise StS (average duration per muscle group and per limb was 86–314 s) on measures of muscle strength and power in non-athletic (i.e., physically inactive and recreationally trained) and athletic (i.e., competitive) participants. They reported that StS negatively affected maximal strength (∆5.4%) and power (∆1.9%) performances irrespective of the participant’s age, gender, or fitness level. According to these results, the authors recommended not to apply StS during a warm-up routine. StS-related performance declines have comprehensively been reported in the form of a position statement from the European College of Sport Sciences (Magnusson and Renström, 2006). These authors concluded that there is ample evidence to suggest that StS should be avoided before activities that require maximal efforts (e.g., maximal strength-, power-related tasks such as jumping). Furthermore, guidelines from the American College of Sports Medicine recommended not to include StS as an integral part of a warm-up routine (Garber et al., 2011).

### Recent Evidence on the Acute Effects of Static Stretching on Muscle Strength and Power

#### Dose-Response Relations

Several original articles, systematic reviews, and meta-analyses examined the acute effects of StS on strength- and power-related actions over the past years (Kay and Blazevich, 2012; Behm et al., 2016; Palmer et al., 2019). Recently, Palmer et al. (2019) conducted a randomized, crossover repeated measures study design in young healthy female participants (21 years) that examined the acute effects of different StS durations (i.e., 30, 60, and 120 s) of the hamstrings on maximal strength and power (i.e., rate of torque development). These authors observed significant declines in muscle power 120 s post-StS but not after 30 and 60 s. Caldwell et al. (2019) investigated the acute effects of prolonged unilateral hamstrings StS (120 s) on knee extension strength (i.e., maximum voluntary isometric contraction) of ipsilateral and contralateral legs and drop jump characteristics (i.e., ground contact time and jump height) in a sample of 40 participants including trained and recreationally active males (*n* = 22) and females (*n* = 18) aged 20–47 years. They revealed a significant performance decline in knee extensor strength after StS in both, the ipsilateral (∆ − 8%) and contralateral (∆ − 4.2%) leg. Pulverenti et al. (2019) examined the acute effects of long-duration (5 × 60 s) StS of plantar flexor muscles on maximal voluntary isometric torque in healthy male subjects aged 28 years. Authors observed a significant decrement in maximal voluntary plantar flexion torque after StS. In their systematic literature review, Kay and Blazevich (2012) examined 106 StS studies and demonstrated that ≥60 s of StS per muscle group resulted in an average performance decline of 7.5% in measures of muscle strength. However, the same authors demonstrated that StS for less than 45 s can be used during warm-up routines without any significant risk of harmful effects on strength and power performances. Four years later, Behm et al. (2016) identified 125 studies in their systematic review which examined the acute effects of StS on strength- and power-related performance measures in physically active and competitive participants. In line with Kay and Blazevich (2012), they demonstrated that ≥60 s of StS per muscle group substantially inhibits strength and power measures (∆4.6%). Alternatively, StS totaling ≤60 s has proved to be less harmful (∆1.1%) (Behm et al., 2016). Overall, the negative acute effects of StS have to be interpreted from a dose-response perspective. In other words, StS conducted over short durations (≤60 s per muscle group) can be recommended while long-duration (≥60 s per muscle group) StS has negative effects on strength and power performances (Kay and Blazevich, 2012; Behm et al., 2016; Caldwell et al., 2019; Palmer et al., 2019; Pulverenti et al., 2019). These findings contradict the widespread opinion that StS inhibits performance in strength- and power-related activities. Recent evidence illustrates that it is primarily a matter of total stretching duration (Kay and Blazevich, 2012; Behm et al., 2016; Caldwell et al., 2019; Palmer et al., 2019; Pulverenti et al., 2019).

#### Static Stretching as an Integral Part of a Full Warm-Up Routine

A major issue that may confound the external validity of the previous studies is that StS was mostly applied in these studies as a single-mode intervention or in the form of an isolated component during a warm-up program. However, this is a rather laboratory-based or artificial form of StS application. In training practice, StS is most often part of an integrated full dynamic warm-up program (Taylor et al., 2009; Blazevich et al., 2018; Reid et al., 2018). Recently, Reid et al. (2018) examined the effects of different StS durations (i.e., 30, 60, or 120 s) of the knee flexors (hamstrings) and extensors (quadriceps) as part of a full warm-up practice (aerobic activity, dynamic stretching, sport-specific activities) on muscle strength and power (i.e., jump height) in physically active male participants aged 27.6 years. The authors revealed that while all stretch durations improved ROM, clear reductions in strength and power measures were found with 120 s of StS per muscle group. However, ≤60 s of StS per muscle group resulted in increased ROM and either no change or beneficial effects on strength and power performances (Reid et al., 2018). The same authors suggested to include StS in a pre-exercise warm-up program because it has the potential to lower the risk of sustaining musculotendinous injuries (Woods et al., 2007; Behm et al., 2016). Additionally, Bengtsson et al. (2018) studied whether sport-specific exercises could restore the negative effects of StS (>60 s) on peak torque of the knee extensors in active physical education male (24.2 years) and female (23.6 years) students. These authors demonstrated no negative effects of StS on isokinetic muscle performance when followed by sport-specific exercises (Bengtsson et al., 2018). Furthermore, in a randomized controlled crossover study, Blazevich et al. (2018) examined the effects of short- (5 s) and moderate (30 s)-duration StS of selected upper and lower limb muscles (e.g., calves, quadriceps, hamstrings, hip flexors and adductors, gluteals, upper chest, and shoulders) as part of an entire warm-up routine (aerobic activity, dynamic activities, and sport-specific activities) on for instance, muscle power (i.e., jump height) in male team sport athletes aged 21.1 years. These authors observed no negative effects of short-duration StS on power performance. Accordingly, and with reference to previous findings (Witvrouw et al., 2004; Woods et al., 2007; McHugh and Cosgrave, 2010; Behm et al., 2016) of small-to-moderate reductions in muscle injury rate in running- and change of direction-based sports, Blazevich et al. (2018) recommended the use of short-duration StS as an integral part of a pre-exercise warm-up routine that includes aerobic activity, dynamic activities, and sport-specific activities. Furthermore, participants in the Blazevich et al. (2018) study experienced positive psychological benefits expressing that they were more likely to perform well when stretching was performed as part of the warm-up, irrespective of the stretch type. A positive psychological outlook is an important component of optimal performance.

### Underlying Physiological Mechanisms Following Longer Duration (>60 s per Muscle Group) Static Stretching

#### Central Mechanisms

It has previously been shown that the neural system is affected by longer duration StS (Trajano et al., 2017). For instance, Avela et al. (1999) reported declines in maximal voluntary activation of the triceps surae muscle following >60 s of StS in healthy male participants aged 21–44 years. Likewise, Fowles et al. (2000) observed decreases in motor unit activation of the plantar flexors following long-duration (>60 s) StS in a sample including recreationally active young men (22.3 years) and women (20.3 years). These modulated neural mechanisms are likely to be associated with the observed decrease in strength and power performances (Avela et al., 1999; Fowles et al., 2000). Additionally, Marek et al. (2005) showed decreases in motor unit activation [lower electromyographic (EMG) amplitude] of the vastus lateralis and rectus femoris muscles at slow (60° s^−1^) and fast (300° s^−1^) velocities following long-duration (>60 s) StS in recreationally active male (21 years) and female (23 years) participants. Further, Trajano et al. (2013) observed long-duration (>60 s) StS-induced lower activation of the plantar flexors in healthy male participants aged 26.5 years. The decrease in muscle activation was illustrated by a reduction in EMG amplitude and V-wave (a variant of the H-reflex providing insight into the voluntary drive to the motoneurons) (Trajano et al., 2013). Recently, Palmer et al. (2019) studied the effects of long-duration (120 s) StS on neural activation of the hamstrings in healthy females aged 21 years. The authors revealed that the rate of EMG rise (i.e., rate of muscle activation) was significantly affected by long-duration (>60 s) StS (Palmer et al., 2019). Mitchell et al. (2011) reported that the rate of EMG rise is influenced by factors that involve early recruitment of motor units, discharge rates, and rate of doublet discharge. Accordingly, it appears logical to state that the decreases in the rate of EMG rise after long-duration StS is due to potential impairments in these physiological characteristics. Caldwell et al. (2019) investigated the acute effects of prolonged unilateral hamstrings StS (120 s) on EMG activity during maximum voluntary isometric contraction of knee extension of ipsilateral and contralateral legs in trained and recreationally active males (*n* = 22) and females (*n* = 18) aged between 20 and 47 years. The same authors reported no changes in EMG activity for either leg after StS. The difference between the study of Caldwell et al. (2019) and the previous detailed research appears to be related to the applied StS protocols. Particularly, in the Caldwell et al. (2019) study the agonists (hamstrings) were stretched and the effects were examined in the antagonist (quadriceps) and contralateral muscles. Pulverenti et al. (2019) examined the acute effects of long-duration (5 × 60 s) StS of plantar flexor muscles on corticospinal excitability and EMG activity of the triceps surae in healthy male subjects aged 28 years. The authors demonstrated that motor-evoked potential, which is elicited using transcranial magnetic stimulation and used as an estimation of corticospinal excitability remained unchanged after StS. Authors concluded that long-duration StS does not alter corticospinal excitability. Unlike corticospinal excitability, significant decrease in EMG activity of the triceps surae following StS was reported (Pulverenti et al., 2019). More conclusive evidence on the principle central mechanisms underpinning declines in strength and power performance following long-duration (>60 s) StS is needed.

#### Peripheral Mechanisms

In terms of peripheral mechanisms, there is compelling evidence that StS affects the MTU. Among the main physiological factors that have been suggested to explain altered muscle functioning following acute StS are changes in viscoelastic properties of the MTU which result in increased MTU compliance and a subsequent decrease in MTU stiffness (Rubini et al., 2007; McHugh and Cosgrave, 2010; Behm and Chaouachi, 2011; Kallerud and Gleeson, 2013). This may impair performance of tasks conducted in the stretch-shortening cycle (SSC) (Kallerud and Gleeson, 2013). Increased MTU compliance could lower the elastic potentiation produced during the stretch phase of SSC activities. Further, greater MTU compliance may additionally affect the length-tension relationship of the muscle which compromises the force-generating capacities (Rubini et al., 2007; Kallerud and Gleeson, 2013). Matsuo et al. (2013) studied the effects of long-duration StS (i.e., 180, and 300 s) on the hamstrings’ contractile properties (e.g., stiffness) in healthy male and female participants aged 20 years. These authors reported significant decreases in MTU stiffness 300 and 180 s post-StS. The decrease in MTU stiffness partially contributes to lower the muscles’ capacity to generate torque (Matsuo et al., 2013). Recently, Nojiri et al. (2019) demonstrated that the stiffness of the iliacus muscle decreased after 60 s of StS and further decreased after 5 min of StS in healthy male participants aged 23 years. Also, Konrad et al. (2019) examined the time course of the changes in MTU mechanical properties of the gastrocnemius medialis after 5 min of StS in healthy male and female participants aged 25 years. These authors observed a decrease in muscle stiffness immediately (i.e., 0 min) and 5 min following StS but not 10 min post-StS.

### Underlying Physiological Mechanisms Following Short-Duration (≤60 s per Muscle Group) Static Stretching Performed as a Single-Mode Treatment or When Integrated Into a Full Warm-Up Routine

The physiological mechanisms underlying longer duration StS have been extensively examined (Trajano et al., 2013, 2014; Behm et al., 2016). However, less is known about the potential mechanisms underpinning short-duration StS when performed as a single-mode treatment or when integrated into a full warm-up routine.

#### Single-Mode Short-Duration (≤60 s per Muscle Group) Static Stretching

##### Central Mechanisms

Regarding central mechanisms, Kay et al. (2015) explored the effects of short-duration StS (60 s) on peak triceps surae EMG activity during a maximal isometric contraction in recreationally active males and females aged 25.6 years. These authors demonstrated no significant changes in muscle activation following short-duration StS. In the same context, Palmer et al. (2019) studied the effects of short StS durations (30 and 60 s) on neural activation of the hamstrings in healthy females aged 21 years. The authors reported that the rate of EMG rise (i.e., rate of muscle activation) was not significantly affected by short-duration (≤60 s) StS (Palmer et al., 2019). Given that the rate of EMG rise is moderated by factors that comprise early recruitment of motor units, discharge rates, and rate of doublet discharge (Mitchell et al., 2011), it appears plausible to argue that after short-duration (≤60 s) StS, these aspects seem not to be significantly affected (Palmer et al., 2019). More research in this area is needed to verify these preliminary results.

##### Peripheral Mechanisms

On the peripheral level, Matsuo et al. (2013) studied the effects of short-duration (i.e., 20 and 60 s) StS on the hamstrings’ contractile properties (e.g., stiffness) in healthy male and female participants aged 20 years. Authors showed no significant changes in muscle stiffness after 20 and 60 s of StS. The unchanged MTU stiffness associated with short-duration StS could contribute to maintaining the capacity of the muscles to generate torque (Matsuo et al., 2013). In the same context, Palmer et al. (2019) revealed that 30 and 60-s StS stretching resulted in only minor reductions in MTU stiffness with no detrimental effects on rate of torque development in healthy females aged 21 years. Kay et al. (2015) examined the effects of short-duration StS of ankle dorsiflexors on Achilles and gastrocnemius medialis stiffness in recreationally active male and female participants aged 25.6 years. They revealed that short-duration StS significantly reduced muscle but not tendon stiffness and concluded that stiffness alteration following short-duration StS seems to be tissue-specific.

#### Static Stretching as an Integral Part of a Full Warm-Up Routine

##### Central Mechanisms

To the best of the authors’ knowledge, studies on the central mechanisms of short-duration StS as an integral part of a full warm-up routine are scarce. Recently, Reid et al. (2018) examined the effects of different StS durations (i.e., 30, 60, or 120 s) as part of a full warm-up practice on the activation of the vastus lateralis and biceps femoris in male physically active participants aged 27.6 years. The same authors reported no changes in EMG activity following any StS condition although strength and power measures were affected by prolonged StS (i.e., 120 s). The disparity between muscle activation (i.e., EMG) and performance (e.g., strength) seems to be due to the poor sensitivity of EMG to changes in muscle activation when measured at very high force levels (Reid et al., 2018). In addition, Reid et al. (2018) reported that irrespective of the StS duration, a decrease in the percentage of voluntary activation was observed. Interestingly, despite these StS-inducing neuromuscular activation impairments, muscle strength and power seem not to be concurrently affected by short-duration StS (i.e., 30 and 60 s) while prolonged StS (i.e., 120 s) leads to significant decreases in muscle strength and power (Reid et al., 2018). Given the lack of as well as the inconsistencies between physiological and performance measures, future studies should be conducted to identify more conclusive evidence.

##### Peripheral Mechanisms

Considering the peripheral mechanisms, during dynamic warm-up, muscles are stretched (contracted) actively through a variety of dynamic activities (Taylor et al., 2009; Blazevich et al., 2018; Reid et al., 2018), increasing body and muscle temperature (Bishop, 2003). In this regard, it has been shown that increased muscle temperature caused by warm-up is accompanied by increased muscle fiber conduction velocity (Pearce et al., 2012) and improved binding of contractile proteins (actin, myosin) (Sale, 2002). Furthermore, a large positive association between muscle temperature and power output has been reported (Bergh and Ekblom, 1979; Sargeant, 1987; Racinais and Oksa, 2010). Racinais and Oksa (2010) showed that a 1°C increase in muscle temperature was accompanied by 2–5% improvement in muscle power performance. Additionally, it has been reported that elevated muscle temperature alters the force-velocity relationship by ultimately allowing higher power output in healthy participants aged 21 years (De Ruiter and De Haan, 2000). From a metabolic point of view, it has been demonstrated that an increase in muscle temperature results in greater phosphocreatine and adenosine triphosphate utilization and higher maximal power output (Gray et al., 2008). However, future studies are needed to confirm the aforementioned mechanisms.

## Can Recent Evidence Clarify Previous Controversies?

**Figure 1:**
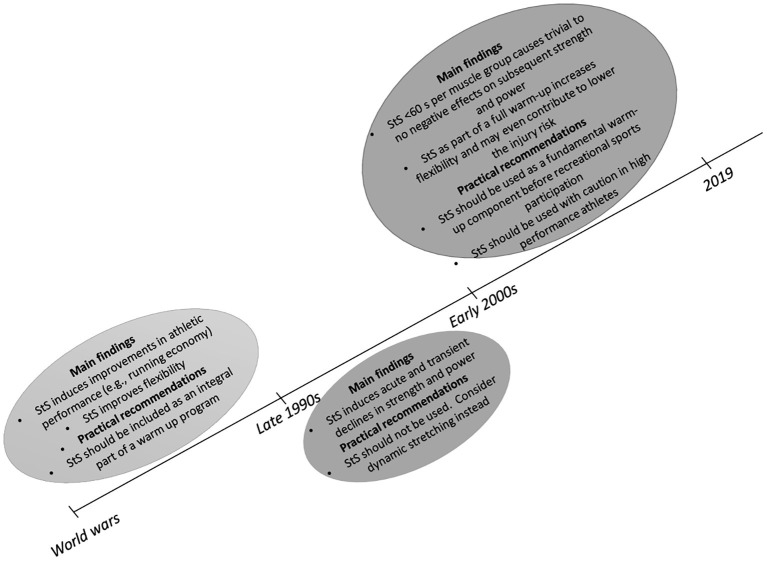
Timeline of the controversial mindset about the acute effects of static stretching (StS) on strength and power performances.

The literature on StS has been subject to controversial debate over the past decades (Figure 1). The general belief that dominated the last two decades is that StS not only contributes to the prevention of injuries (Thacker et al., 2004; Witvrouw et al., 2004) but also impairs athletic performance (Pope et al., 2000; Shrier, 2004a,b; McHugh and Cosgrave, 2010; Simic et al., 2013). Accordingly, it has been recommended not to apply StS in a pre-exercise warm-up routine, especially if strength- and power-related activities are performed subsequently (Magnusson and Renström, 2006; Garber et al., 2011; Simic et al., 2013). However, more recent evidence suggests a differentiated view on the effects of StS, which is based on established dose–response relations (Kay and Blazevich, 2012; Behm et al., 2016; Palmer et al., 2019). In particular, it has been demonstrated that short-duration StS (≤60 s per muscle group) can be used with trivial negative risks on subsequent strength- or power-related tasks. However, long-duration StS (>60 s per muscle group) can cause substantial and practically relevant decrements in strength and power performances. In addition, recent evidence suggests that when considered within a full warm-up routine, short-duration StS may even contribute to lower the risk of sustaining musculotendinous injuries (Small et al., 2008; Blazevich et al., 2018; Reid et al., 2018). In fact, Witvrouw et al. (2004) suggested that a sufficient level of MTU compliance is needed for sports conducted in the SSC to effectively store and release a high amount of elastic energy. In case of insufficient MTU compliance, the demands in energy absorption and release may rapidly exceed the capacity of the MTU, which may cause a higher risk of injuries (Witvrouw et al., 2004). Having these findings in mind, it is timely and imperative to revise recommendations on StS. Clearly, short-duration (≤60 s per muscle group) StS can be performed as part of a full warm-up routine before strength- and power-related activities with negligible risk of performance harm and a potentially positive impact on flexibility and musculotendinous injury occurrence in physically active individuals. However, in high-performance athletes, short-duration StS has to be applied with caution in particular before competition due to its slightly negative but still prevalent effects on subsequent strength and power performances.

## Conclusions and Practical Applications

There is strong evidence suggesting that StS causes only trivial negative effects on subsequent strength and power performances if the accumulated duration per muscle group does not exceed 60 s. Consequently, we should update previous statements on the harmful effects of StS on strength and power performances. Overall, coaches are advised to consider short-duration StS as an important warm-up component in recreational sports due to its potentially positive effect on flexibility and musculotendinous injury prevention. However, in high-performance sports, minimum performance differences can have a major impact on athletes’ success in competition. Given the trivial negative effects of short-duration StS on subsequent strength and power performances, StS should be applied with caution in high-performance athletes. The underlying mechanisms of short-duration StS when performed as a single-mode treatment or when integrated into a full warm-up practice have to be substantiated by future empirical studies. Additionally, the chronic effects of StS on muscle strength and power as well as injury prevention should be a focus in future research.

## Author Contributions

All authors listed have made a substantial, direct and intellectual contribution to the work, and approved it for publication.

### Conflict of Interest

The authors declare that the research was conducted in the absence of any commercial or financial relationships that could be construed as a potential conflict of interest.

The reviewer MC declared a past co-authorship with HC to the handling editor.
